# Scaling up combined community-based HIV prevention interventions targeting truck drivers in Morocco: effectiveness on HIV testing and counseling

**DOI:** 10.1186/s12879-015-0936-6

**Published:** 2015-05-05

**Authors:** Hakima Himmich, Lahoucine Ouarsas, Fatima Zahra Hajouji, Caroline Lions, Perrine Roux, Patrizia Carrieri

**Affiliations:** Moroccan Association for the Fight against AIDS (ALCS), Casablanca, Morocco; INSERM, U912 (SESSTIM), Marseille, France; Aix Marseille University, IRD, UMR-S912, Marseille, France; ORS PACA, Observatoire Régional de la Santé Provence Alpes Côte d’Azur, Marseille, France

**Keywords:** Community interventions, HIV testing, Counseling, Truck drivers, Sexual transmission

## Abstract

**Background:**

Truck drivers constitute an important bridging group in the HIV epidemic in Morocco. This study examined the effect of a community-based educational intervention in Morocco on HIV testing and counseling, in representative samples of truck drivers before (2007) and after (2012) the intervention.

**Methods:**

Face-to-face structured interviews, adapted from UNAIDS documents, collected data on socio-demographic characteristics, HIV testing and counseling, and HIV risk behaviors in both the 2007 and 2012 surveys. Information about exposure to the intervention was also collected in the latter. Individuals exposed to the intervention were compared with those unexposed (i.e. unexposed in 2012, and all the 2007 pre-intervention sample).

**Results:**

The 2012 group included 459 men with a median [IQR] age of 38 [31–44] years, 53% of whom reported exposure to the educational intervention. The percentage of participants tested for HIV and receiving HIV counseling in the last 12 months, was significantly higher in the 2012 group (29.6% vs 4.3% in 2007). Data from the 2012 survey confirmed a significant positive trend between being HIV tested and receiving counseling and the number of times a participant was exposed to the intervention (once: (OR = 5.17(2.38-11.25)), twice or more (OR = 19.16(10.33 - 35.53)). These results were confirmed after adjustment for employment, knowledge that the HIV test results would remain confidential, inconsistent condom use with occasional partners or sex workers, and when including individuals from 2007 considered unexposed.

**Conclusions:**

Community-based educational interventions targeting truck drivers can be effective in increasing coverage of HIV testing and counseling, particularly if they are repeated and cover a considerable portion of this at-risk population. These results are encouraging for other countries which urgently need to implement prevention interventions for most-at-risk populations. Furthermore, they clearly show the power of community-based organization interventions in settings where resources for HIV prevention remain limited.

## Background

Mobility is now considered a major contextual factor fuelling the spread of HIV across different geographic areas [1-3]. Truck drivers have been identified as a group especially vulnerable to HIV and sexually transmitted infection (STI) transmission. The specific context of truck drivers, which combines occupational stress, expanded social contact and sexual networks, exposes them to unsafe sexual behaviors with occasional partners and sex workers [4].

Opportunities of sexual encounters for truck drivers include truck stops, weigh and fuel stations, highway rest and picnic areas, and various off-road establishments (e.g., adult entertainment) [5].

Because truck drivers are a potential bridging group for HIV and STI transmission to the general population, they are targeted for risk reduction interventions in many countries [6-9]. Several studies have consistently documented higher HIV, STI and even HCV prevalence in this group [10], as well as reduced HIV testing and counseling [6,11-13].

The development of rapid HIV testing methods has led to voluntary counseling and testing (VCT) becoming a major tool for HIV prevention for two primary reasons: first, individuals aware of their positive serostatus are less likely to engage in HIV at-risk behaviors. Second, VCT is the first step in a chain reaction which includes HIV care, access to antiretrovirals, controlled viremia and finally reduced likelihood of transmitting the virus [14]. Therefore, increased access to HIV screening is also a health policy challenge as it leads to reduced delays in diagnosis and HIV care and potentially a decrease in the number of new HIV cases.

As is the case in many other countries like India and South-Africa, the role that truck drivers play as a “bridging group” in fuelling the HIV epidemic is a public health concern in Morocco. HIV incidence statistical modeling in Morocco estimated that 67% [15] of new HIV cases occur in the network of professional sex workers and their clients. A “knowledge, attitudes, beliefs and practices” (KABP) survey conducted in 2007 [16] in a random sample of truck drivers in several geographic areas of Morocco, showed that this group was characterized by poor knowledge of HIV and STI transmission, poor levels of VCT and that they engaged in high-risk sexual behaviors with occasional partners and sex workers. We conducted an identical survey in 2012 on a random sample of truck drivers in the same geographic areas, using the same methodology to assess the effect of a continuous community-based outreach educational intervention implemented between 2007 and 2012.

This before-after study provided us with the opportunity to estimate the overall effect of a large-scale community-based intervention on several outcomes, in particular on VCT in the population of truck drivers in Morocco. It also enabled us to assess the effect which multiple exposure to this intervention would have on VCT, after adjusting for potential confounders or correlates.

## Methods

### Ethical considerations

The study was approved by the Moroccan Ethical Committee for Biomedical research. All participants were informed about the study and had to provide written, informed consent to be included. The survey was anonymous.

### Intervention

This study consisted in a before-after analysis of a 5-year public health intervention, implemented between 2007 and 2012 in Morocco. The intervention was based on an outreach approach developed by the main community-based organization (CBO) for the fight against HIV in Morocco (ALCS) in specific areas frequented by truck drivers. It comprised the training of community staff in “knowledge transfer” about HIV prevention and care to those exposed to the intervention, the provision of HIV prevention tools (condoms, educational tools), referral to community HIV and syphilis voluntary counseling testing services, in addition to HIV and STI care support and referral for those who were diagnosed positive.

More specifically, the interventions comprised the following actions:

- Providing information and education aimed at changing sexual behaviors. This was performed by trained field workers twice a week at the sites in the 6 main towns (Agadir, Béni Mellal, Casablanca, Guelmim, Marrakech and Tanger) where truck drivers gathered. During these interventions - scheduled according to an updated geographical map - condoms and information support were distributed and truck drivers were referred to HIV and STI screening services.
- Identifying peers in these 6 gathering areas, in order to assure the continuity of the intervention. The activity of these peers was monitored and supervised by the field workers.
- Referring drivers to HIV screening mobile units, depending on the truck drivers’ demand for HIV screening and for STI care in the different sites.

### Study population

We enrolled truck drivers from shipping companies and other drivers working on the main highways in Morocco between Guelmim and Tanger: Guelmim-Agadir, Agadir-Casablanca and Casablanca-Tanger. Inclusion criteria were as follows: resident in Morocco and spending at least one night away from home while working. The only exclusion criterion was being under 18 years old.

Two “control” groups were defined: the first was the study sample interviewed in 2007 before the implementation of the prevention intervention. The second was the subsample of individuals interviewed in 2012, but who reported they had not been exposed to the intervention.

The first group was used as an “historical” control group in order to evaluate whether scaling-up of the prevention intervention after 2007 had major “population” effects on several outcomes such as knowledge, attitudes and practices, and particularly on VCT.

The second group was used as a control group to verify whether individuals who had been exposed to the intervention one or more times were more likely to have been HIV-tested during the year prior to the 2012 survey (outcome) than those unexposed. We also tested for the existence of a dose–response relationship. Known correlates of receiving HIV-testing and counseling (age, sexual risk behaviors and socio-economic level) and possible confounders were used as adjustment variables.

### Questionnaires and data collection

The questionnaire for the face-to-face interview was adapted from UNAIDS documents used in behavioral surveillance studies and administered by trained and/or experienced interviewers who had not previously been involved in this specific outreach intervention. The standardized questionnaire was drawn up in French and translated into Darija, the colloquial Arabic dialect, to ensure that the studied population would understand its contents more comprehensively. It was pre-tested and validated on a sample of 20 truck drivers and used to collect data on the outcome (exposure to the intervention), sociodemographic data, and data on participant HIV at-risk behaviors during the 12 months prior to the survey:

- Sociodemographic data included age, gender, marital situation, education level and health insurance.
- Professional data included type of truck driver (national, international, assistant driver, mechanic), work location, length of service etc.
- Having being tested for HIV in the 12 months prior to the survey.
- Drug and alcohol use.
- Sexual behaviors including sexual activity, sexual encounters with occasional partners and professional sex workers, non-systematic condom use with occasional partners or sex workers, and sexual encounters with male partners.
- Knowledge of HIV and STI and the symptoms of HIV and STI.
- Exposure (or not) to the specific prevention intervention. This information included the number of times the participant had contacted intervention stakeholders, the information obtained, whether condoms were obtained or not, etc.

Inconsistent condom use was defined as the non-systematic use of condoms with sexual partners of unknown serostatus (occasional partners or sex workers).

### Statistical methods

#### Sampling

In order to obtain a representative sample of truck drivers which would enable us to create before-after intervention comparisons, we used the same sampling approach in the same areas, i.e. self-weighting cluster sampling. Clusters were randomized using a mapping of the different areas where truck drivers frequented. Cluster randomization took into account the average size of clusters in terms of truck drivers and the length of each intervention for each intervention site.

The study was designed on the hypothesis of an expected decrease in at-risk behaviors in this population. In order to be able to detect the expected effect, from the various hypotheses available we had to choose the one which required the largest sample size. This is why we used condom use for the sample size. Given that the 2007 study showed that 78% of truck drivers reported sexual encounters with sex workers during the previous 12 months, and that 37% reported condom use in the most recent sexual encounter with a sex worker, it was hypothesized that the percentage of condom use would reach 50% after the intervention, i.e. in the 2012 study. Accordingly, to detect a minimum increase of 13 points, with a statistical power of 80%, a confidence level of 95% and a sampling design effect of 2, we had to envisage enrolling 460 truck drivers, divided into 46 clusters of 10 truck drivers.

The two study groups enrolled in 2007 and in 2012 constitute a random sample and can be considered geographically comparable.

#### Statistical analysis: before-after comparison

To compare the characteristics of the study samples enrolled in the 2007 and 2012 surveys, we used a Chi-square test as appropriate for categorical variables (Table 1).

**Table 1:** **Socio-demographic and professional characteristics of participants according to the study sample**

	**2007**	**2012**	***P***
	**(N=484)**	**(N=459)**	
**Age category (yrs)**			
≤24	92 (19.0)	23 (5.0)	*<10* ^*-3*^
25-29	106 (21.9)	67 (14.6)	
30-39	165 (34.1)	177 (38.6)	
40 and over	121 (25.0)	192 (41.8)	
**Living in a couple**			
Yes	302 (65.1)	352 (76.9)	*<10* ^*-3*^
No	162 (34.9)	106 (23.1)	
**Education**			
None	114 (23.7)	79 (17.2)	*0.05*
Primary level	180 (37.4)	183 (39.9)	
Secondary level	187 (38.9)	197 (42.9)	
**Employment status**			
Self-employed	112 (23.4)	199 (43.4)	*<10* ^*-3*^
Employee	367 (76.6)	260 (56.6)	
**Health insurance**			
Yes	149 (31.0)	90 (19.6)	*<10* ^*-3*^
No	332 (69.0)	369 (80.4)	
**Male sexual partners**			
Yes	28 (8.3)	47 (10.8)	*0.26*
No	308 (91.7)	389 (89.2)	
**Paid for sex in the previous 12 months**			
Yes	243 (50.2)	291 (63.4)	*<10* ^*-3*^
No	241 (49.8)	168 (36.6)	
**Inconsistent condom use with occasional partners and/or sex workers in the previous 12 months**			
No	29 (6.0)	36 (7.8)	*0.001*
Inconsistent condom use only with occasional partners	32 (6.6)	62 (13.5)	
Inconsistent condom use with sex workers	423 (87.4)	361 (78.6)	
**Knowledge that the results of HIV testing would remain confidential**			
No	349 (76.7)	76 (16.8)	
Yes	106 (23.3)	377 (83.2)	*<10* ^*-3*^
**STI symptoms (penis discharge in the previous 12 months**			
Yes	109 (23.7)	115 (25.3)	*0.29*
No	351 (76.3)	340 (74.7)	
**STI symptoms (ulceration in the previous 12 months)**			
Yes	38 (8.3)	47 (10.4)	*0.29*
No	418 (91.7)	406 (89.6)	
**Poor Knowledge about HIV prevention**			
Yes	296 (61.2)	144 (31.4)	*<10* ^*-3*^
No	188 (38.8)	315 (68.6)	

To identify potential confounders or factors which were presumed to have been modified by the intervention, we compared specific behaviors or knowledge in the study samples in unexposed (2007, 2012) and exposed (2012) using a Chi-square test as appropriate for categorical variables and for sub-group comparisons (Table 2).

**Table 2:** **Before and after a targeted intervention: comparisons of knowledge, HIV sexual risk behaviors and practices**

	**2007**	**2012**					
		**Exposed to the intervention**					
		**Never**	**Once**	**Twice or more**	**Total**	p*	P**
		**(n = 210)**	**(n = 63)**	**(n = 180)**			
Knowledge of HIV prevention methods	38.8%	59.0%	71.4%	79.4%	68.6%	*<10* ^*−3*^	*<10* ^*−3*^
Condom use during the most recent sexual encounter with a sex worker	35.5%	40.5%	68.3%	78.5%	60.3%	*<10* ^*−3*^	*0.34*
Systematic condom use with a sex worker in the previous 12 months	12.8%	10.5%	19.0	35.0	21.4%	*<10* ^*−3*^	*0.38*
Voluntary HIV testing and counselling in the previous 12 months	4.3%	6.7%	27.0%	57.8%	29.6%	*<10* ^*−3*^	*0.20*
Knowledge of non-AIDS STI symptoms (men)	24.6%	25.7%	31.7%	36.7%	30.7%	*0.02*	*0.75*
Knowledge of non-AIDS STI symptoms (women)	13.4%	9.5%	14.3%	20.0%	14.2%	*0.03*	*0.15*
Knowledge that the results of HIV test would remain confidential	23.3%	75.1%	83.9%	92.2%	83.2%	*<10* ^*−3*^	*<10* ^*−3*^

*General comparison.

**Comparison between 2007 and unexposed in 2012.

#### Statistical analysis: correlates of having received HIV testing and counseling in 2012 survey (Table 3)

Focusing only on the 2012 sample, we assessed the existence of a dose–response relationship between the number of times the participant had been exposed to the intervention (once or more than once) and VCT using univariate and multivariate logistic regression analysis to take into account possible confounders.

**Table 3:** **Potential predictors of VCT (logistic regression model)**

	**N**	**VCT in previous 12 months**				
		**%**	**ORc (95% CI)**	**Complete model**	**Final model**	**Sensitivity analysis**
				**ORa (95%) (N=423)**	**ORa (95%) (N=451)**	**ORa (95%) (N=902)**
**Exposed to the intervention**						
2007	484	4.3	**--**	**--**	--	1
Never	210	6.7	1	1	1	0.80 (0.37-1.71)
Once	63	27.0	5.17 (2.38 - 11.25)	5.45 (2.27-13.06)	4.84 (2.18-10.78)	3.81 (1.73-8.36)
Twice or more	180	57.8	19.16 (10.33 - 35.53)	16.65 (8.10-34.23)	15.72 (8.31-29.73)	12.18 (6.54-22.68)
**Age category (yrs)**						
≤24	23	17.4	0.50 (0.16-1.53)	1.94 (0.43-8.81)		
25-29	67	23.9	0.74 (0.39-1.41)	0.98 (0.39-2.45)		
30-39	177	33.3	1.18 (0.76-1.84)	1.30 (0.70-2.42)		
40 and over	192	29.7	1.00	1		
**Living in a couple** (Yes vs. No)	352 vs. 106	30.4 vs.26.4	1.22 (0.75-1.98)	1.30 (0.62-2.71)		
**Education**						
None	79	22.8	0.67 (0.37-1.24)	1.29 (0.54-3.06)		
Primary level	183	31.7	1.06 (0.68-1.64)	1.19 (0.70-2.11)		
Secondary level	197	30.5	1.00			
**Employment status** (self-employed versus employee)	199 vs 260	34.2 vs. 26.2	1.47 (0.98-2.19)	1.71 (0.99-2.96)	1.67 (1.02-2.73)	1.44 (0.93-2.22)
**Health insurance** (Yes versus No)	90 vs 369	28.9 vs. 29.8	0.96 (0.58-1.59)	1.12 (0.56-2.24)		
**Male sexual partners** (Yes vs. No)	47 vs. 389	48.9 vs. 27.8	2.49 (1.35-4.61)	2.47 (1.12-5.46)		
**Paid for sex in the previous 12 months** (Yes vs. No)	291 vs. 168	30.6 vs.28.0	0.88 (0.58-1.34)	1.61 (0.77-3.33)		
**Inconsistent condom use with occasional partners and/or sex workers in the previous 12 months**						
No	36	47.2	4.28 (2.45-7.47)	3.62 (1.64-7.98)	3.07 (1.59-5.93)	2.77 (1.54-4.97)
Inconsistent condom use only with occasional partners	62	56.5	2.95 (1.47-5.93)	1.43 (0.57-3.57)	1.16 (0.53-2.54)	1.41 (0.70-2.87)
Inconsistent condom use with sex workers*	361	23.3	1.0	1.0	1.0	1.0
**STI symptoms: penis discharge in the previous 12 months** (Yes vs. No)	115 vs. 340	18.3 vs.33.5	0.44 (0.26-0.75)	0.73 (0.33-1.59)		
**STI symptoms: ulceration in the previous 12 months** (Yes vs. No)	47 vs. 406	27.7 vs. 29.6	0.91 (0.47-1.79)	1.67 (0.61-4.59)		
**P Poor Knowledge about HIV prevention** (Yes vs. No)	144 vs. 315	22.2 vs. 33.3	0.58 (0.37-0.92)	0.81 (0.43-1.56)		
**Knowledge that the results of HIV test would remain confidential**	377 vs. 76	34.0 vs. 10.5	4.37 (2.04-9.37)	2.84 (1.12-7.24)	2.99 (1.24-7.21)	3.01 (1.61-5.61)

The following correlates and possible confounders taken into consideration were: main socio-demographic characteristics (age, education (primary, secondary level vs. none), living in a couple, health insurance, employment status (self-employed versus employee)), sexual behaviors (sexual relationships with men, inconsistent condom use with the most recent sexual partner and/or the most recent occasional sexual partner, specific IST symptoms), knowledge that the results of HIV test would remain confidential and number of times the participant had been exposed to the community intervention (never, at least once, more than once).

To assess the independent effect of the intervention on VCT, using a backward procedure based on the likelihood ratio test with a p-to stay = 0.05, we built the final model, entering all possible confounders previously identified and correlates with a p value <0.20 [17]. Finally, we ran the same analysis (sensitivity analysis) on an extended dataset also including the 2007 study group (used as an additional unexposed group) to test the robustness of the results and the association between the intervention and VCT (Table 3).

All analyses were performed using SPSS 17.0.

## Results and discussion

### Characteristics of the study group

The characteristics of the 2007 and 2012 study samples are described in Table 1.

The 2012 group enrolled 459 men with median [IQR] age of 38 [31–44] years. Seventeen percent and 40% had no formal educational or only primary education, respectively. More than two thirds (77%) reported they were married or living in a couple and only 20% reported they had health insurance. The comparisons shows the significant evolution of the characteristics of truck drivers in the two study groups (Table 1), which are both representative of the population of truck drivers in 2007 and 2012. The variables which significantly differed between the two groups were considered potential correlates/confounders in the analysis of the relationship between the intervention and VCT.

### Overall effect of the intervention in the targeted population

The proportion of those who received HIV testing and counseling in the 12 months prior to the 2012 survey varied considerably, depending on the number of times the participants had been exposed to the intervention: 6.7% for those who had not been exposed to the intervention, 27.0% in those who had been exposed once and 57.8% in those who had been exposed more than once (Table 2). A dose–response relationship in the 2012 survey is clearly observed with increasing levels of VCT in individuals who reported benefiting one or more times from the intervention (p < 10^−3^). It is important to point out that the prevalence of VCT in those who had not been exposed to the intervention in 2012 (i.e. the control group) was comparable to that in the sample in 2007 (i.e. the historical control group).

Table 2 reports also a comparison of main indicators presumed to be modified by the intervention (knowledge of HIV prevention methods, condom use during the most recent sexual encounter with a sex worker, systematic condom use with a sex worker in the previous 12 months, VCT in the previous 12 months, knowledge of non-AIDS STI symptoms in men, and knowledge of non-AIDS STI symptoms in women) in individuals exposed (2012) compared with unexposed in 2012 and with the historical unexposed control group (2007). A significant difference was found for all indicators between those exposed and those unexposed to the intervention, but percentages are comparable between the historical unexposed group and the unexposed group in 2012 (Table 2) except for knowledge about HIV prevention and about the confidentiality of the HIV test result, which were significantly higher in the latter group.

### Factors associated with VCT

Table 3 reports associations between exposure to the intervention or other potential correlates and/or confounders and having received HIV testing and counseling during the 12 months before the 2012 survey. No major association was found between the socio-demographic variables identified as potential confounders in Table 1 and the outcome. Moreover these variables were not confounders of the association between exposure to the intervention and the outcome, as they did not modify the estimates of OR of such association. Accordingly, they were removed from the multivariate model. By contrast, inconsistent condom use with sex workers was independently and negatively associated with VCT, as well as the employment status and knowledge that the results of the HIV test would remain confidential. A strong dose–response relationship between exposure to the intervention and receiving HIV testing and counseling in the previous 12 months was observed. Compared with those who had never been exposed to the intervention, individuals exposed to it more than once were 19 times more likely to have received HIV testing and counseling. These results were confirmed after adjustment for employment and knowledge that the results of the HIV test would remain confidential and inconsistent condom use with occasional partners or sex workers (Table 3).

The sensitivity analysis, which included data of the 2007 survey, confirmed the major effect of the intervention after adjustment for the same pattern of predictors. The unexposed group in 2012 was not significantly different from the historical unexposed group. Furthermore the dose–response relationship was also confirmed in this analysis. Apart from inconsistent condom use and knowledge that the results of HIV testing would remain confidential, the potential confounders identified in Table 1 were not significantly associated with the outcome and did not modify the relationship between exposure to the intervention and the outcome.

### Discussion

This study is the first in the field of HIV prevention to document risk behaviors in a representative sample of truck drivers in Morocco and show the feasibility and effectiveness of scaling up targeted educational interventions for this population. The main result of the study can be interpreted at two levels. The first concerns the population effect which was observed in two repeated representative samples of truck drivers (before-after study): a 7-fold increase in the coverage of truck drivers who received HIV testing and counseling in the prior 12 months was observed. Most indicators of effectiveness significantly improved between 2007 and 2012, in particular the prevalence of VCT. Interestingly, this prevalence in individuals in the 2012 study sample who had never been exposed to the intervention, was comparable with that measured in the representative pre-intervention sample of truck drivers in 2007 as the sensitivity analysis shows. This would suggest that without this intervention, behaviors would have not changed.

The second level of interpretation is interesting when one considers that a dose–response “exposure to the intervention” relationship was revealed: being exposed to the intervention once, encouraged a considerable proportion of truck drivers to go for VCT. The estimated likelihood of VCT in individuals exposed more than once to the intervention, was approximately 20 times higher than that observed in those who had never been exposed to it, and 4 times higher than that for those who had been exposed only once.

One important predictor was the knowledge that the HIV test results would remain confidential. It is worth noting that the increased percentage of those who had been HIV tested and received counseling in 2012 may be attributable to the increased proportion of truck drivers (23% in 2007 versus 83% in 2012) who realized that the results of the HIV test were confidential.

The global result of the intervention holds, even after inclusion of the historical control group and adjustment for potential correlates and confounders. The effect of the educational intervention seems to be the first step in the causal path leading to greater knowledge about HIV prevention and greater awareness of the test being confidential, and therefore a change in behaviors regarding HIV testing.

Although individuals reporting sex with male partners were twice as likely to report VCT, this association was not confirmed in the multivariate analyses. Individuals reporting inconsistent condom use with sex workers were less likely to report HIV-testing than those reporting consistent condom use or inconsistent condom use with occasional partners. This highlights the need for prompt, tailored and combined interventions for this group where the risk of sexual transmission of HIV is still underestimated. Previous studies have already underlined the effectiveness of sexual risk reduction interventions targeting female sex workers which potentially have an indirect effect on their clients (e.g. the decision to use condoms, or to go for testing, etc.) [18,19]. The perceived stigmatization of HIV-positive individuals, be they sex workers [20] or truck drivers [21], is an important barrier to condom use.

One major correlate of VCT in our study was the knowledge that the results would remain confidential. This is a major issue in many countries, and in Morocco in particular, where HIV-infected individuals (but also other specific groups at risk of HIV transmission, including MSM and drug users) suffer from HIV-based discrimination [22]. Knowledge about the confidentiality of HIV testing results is therefore the primary lever to use when proposing HIV screening to at-risk populations.

Individuals who were self-employed were more likely to have been tested for HIV and to have received counseling than those not self-employed. As self-employment is a proxy of high socio-economic status, this association confirms previous studies which show that low socioeconomic levels are associated with less knowledge about HIV and less VCT [23,24]. On the other hand, individuals who are not self-employed are at higher risk of stigma if they test positive for HIV, and this may be a barrier to going for testing.

Some limitations need to be acknowledged. First certain explanatory variables, like sexual behaviors based on self-reports, may have been affected by social desirability bias. For this reason it is possible that certain HIV risk exposure variables or proxies of HIV exposure (e.g. inconsistent condom use with a sex worker or specific STI symptoms) may have been underestimated in our study group. This may have affected the association between such variables and VCT in a conservative way (i.e., underestimating the strength of the association). However, this bias was reduced by the specific training given to the interviewers and the use of questionnaires which had been previously tested for their consistency. It is important to underline the high external validity of the study population, as the rigorous sampling plan enabled us to obtain a highly representative sample of the population of truck drivers in the target geographic area. In addition, internal validity of the pre-post intervention study was also high as the same sampling plan methodology was used for pre- and post-intervention studies. In order to confirm the robustness of the results and to include an additional unexposed group, we conducted a sensitivity analysis including the two surveys which showed that unexposed individuals - whether unexposed in 2012 or because they were interviewed before the intervention - had a similar likelihood of not being HIV-tested.

## Conclusions

This study clearly shows that this type of intervention is not only feasible but is also effective in a population presenting a high risk of HIV infection. Repeated educational interventions can be particularly beneficial in increasing coverage of HIV testing and counseling.

Their evaluation is necessary to identify subgroups which are less sensitive to this intervention and which may require additional interventions using other entry points (e.g. sex workers using condoms more systematically with their clients). Finally, these results are encouraging for other countries which urgently need to implement prevention interventions for most-at-risk populations and clearly show the power of community-based organization interventions in settings where resources for HIV prevention remain limited.

## Abbreviations

ALCS: Association for the fight against AIDS
CI: Confidence interval
HCV: Hepatitis C virus
HIV: Human immunodeficiency virus
INSERM: Institut National de la Santé et de la Recherche Médicale
IQR: Interquartile range
IRD: Institut de Recherche pour le Développement
KAPB: Knowledge, attitudes, beliefs and practices
OR: Odds ratio
ORS PACA: Observatoire Régionale de la Santé Provence-Alpes-Côte d’Azur
STI: Sexually transmitted infections
UMR: Unité Mixte de Recherche
UNAIDS: United Nations & AIDS
VCT: Voluntary counseling and testing
